# Impact of different components of the Skyrme nucleon–nucleon effective interaction on the nuclear density distribution

**DOI:** 10.1038/s41598-023-44780-6

**Published:** 2023-10-16

**Authors:** W. M. Seif, A. S. Hashem

**Affiliations:** Department of Physics, Faculty of Science, Cairo University, Giza, 12613 Egypt

**Keywords:** Physics, Nuclear physics

## Abstract

We systematically investigate the impact of the different terms of the Skyrme energy density functional of the effective nucleon-nucleon interaction, and of its associated nuclear matter (NM) properties, on the density distributions of spherical nuclei. Twenty five Skyrme force parameterizations are examined simultaneously, covering a broad range of each characteristic parameter and NM property. The diffuseness and the neutron-skin thickness are found to be the most sensitive density quantities to the force parameterization. The diffuseness is indicated to decrease with increasing the central zero-range and the effective mass terms of the effective force, and the power σ of its density dependent term, as well as with the coefficient of the NM symmetry energy (*a*_*sym*_) and its density slope (*L*) at saturation density, and the incompressibility (*K*_*o*_). In contrast, the proton and neutron diffuseness tend to increase with increasing the spin–orbit force and the isoscalar effective nucleon-mass (m*), and to increases slightly with the density dependence parameters other than the power σ. Opposite impacts are pointed out for the different parts of the finite-range, and J^2^ tensor terms on the proton and neutron density. While the neutron-skin thickness tends to increase significantly upon increasing the central zero-range and spin–orbit force terms, *a*_*sym*_, *L,* and *K*_*o*_, and to increase slightly with the finite-range and J^2^ tensor terms, and σ, it decreases with the effective-mass term, the density-dependence exchange parameter, and with the indicated isoscalar effective mass. The proton and neutron radii exhibit decreasing behavior with the central zero-range and the spin–orbit terms, and with *K*_*o*_, and m*. Increasing *a*_*sym*_ and *L* indicate slightly less (larger) proton (neutron) radius.

## Introduction

The density distribution of finite nuclei is mainly determined by the nucleon-nucleon (*NN*) interaction and its different contributions. While the central part of the NN force and the bulk properties of nuclear matter controls the proton and neutron internal densities as they influence the equilibrium saturation density^1,2^, its density-dependence and surface properties determine the corresponding radii and diffuseness^3^. For instance, the saturation density of asymmetric nuclear matter increases upon increasing its incompressibility^4^. The values of the density diffuseness, and its anisotropy and polarization, rely on the characteristics of the NN interaction and nuclear matter (NM) at low sub-saturation densities^3^. This is related also to the allowed maximum isospin asymmetry value for bound asymmetric nuclear matter^2^, where the local isospin-asymmetry increases at the tail density region of neutron rich nuclei. Increasing the isospin-asymmetry within the surface and tail region makes the nuclear matter more soft with respect to the internal region^1,4^. Indeed, the nuclear surface region plays a crucial role in both structure and reactions of nuclei. The density distributions of normal and exotic nuclei provide a reliable source of information on their surface and spectroscopic properties, such as the spin–orbital splitting^5^, the proton and neutron separation energies^6^, the single-particle level distribution, and the related shell structure^5^, in addition to the proton- and neuron-skin thickness. Moreover, the nucleon density profiles of the interacting nuclei affect the formed Coulomb barrier height and radius, and completely determine its curvatures and diffuseness^7–10^, where it is correlated with the ratio of surface to internal nucleons and the occupation probability in various orbits with low and high orbital angular momenta^11^. This directly impacts the α^12–14^ and cluster decays of the nuclei, fusion^15^ and charge exchange^16^ cross-sections, elastic and inelastic diffraction^17^, quasi-elastic excitation function and related barrier distributions^18^, and other nuclear reactions.

Considering the correct nuclear density is then crucial in studying the nuclear structure, reactions, and decays of stable and exotic nuclei. In practice, this concern faces the problem that the calculated density distributions of finite nuclei shows strong model dependence when they derived based on effective NN interaction, far away the availability of experimentally determined charge density of hundreds of nuclei. For instance, the correlation between the neutron skin thickness of mirror nuclei with the charge radii differences and some stellar observables were investigated based on the coupled-cluster theory^19^ and based on finite range simple effective interaction (SEI)^20^. The proton and neutron skins of mirror nuclei were also investigated using high-precision chiral few-nucleon interactions^21^. On the other hand, the effects of the symmetry energy and its slope on the neutron skin thickness of asymmetric semi-infinite nuclear matter and on the neutron skins of Ca, Sn and Pb isotopes were studied using mean-field approximation based on the Hugenholtz–Van Hove theorem^22^ and also in a Bayesian framework^23^. The dependence of the charge radii of mirror nuclei on the derivative of the neutron EOS and on the symmetry energy at saturation density has been used for constraining the neutron EOS to be used in astrophysics^24^. Moreover, the mass and isospin dependence of the symmetry energy and surface properties of even-even Ni, Sn, and Pb nuclei, including their exotic isotopes, have been studied^25^ in the framework of the coherent density fluctuation model for finite nuclei, with Skyrme NN interaction, where more systematic analysis was suggested for the isotopic sensitivity of the neutron skin thickness dependence on the symmetry energy^25^. The studies based on both Skyrme Hartree–Fock and relativistic mean field models confirmed the correlation between the neutron skin thickness and the symmetry energy coefficients for both stable and unstable nuclei^26^. Available data on the neutron skin thickness of Sn isotopes and on the isospin diffusion of heavy-ion collisions at intermediate energies have been combined to obtain more stringent limit for the symmetry energy and its density slope at saturation density^27^. Although the general behaviours associated with investigated quantities do not change much in the above-mentioned studies based on the different models, but the calculated densities exhibit clear model dependence.

In addition to the kinetic and Coulomb contributions, the Skyrme effective interaction consists of six terms, namely the zero-range, density-dependent, effective-mass, and finite-range terms, which form its central part, as well as its spin–orbit and tensor parts. The parameters of these terms are usually adjusted by fitting to nuclear structure data such as masses, charge radii, neutron- and proton-skin thickness, and giant resonances, with constraints on and bulk properties of nuclear matter, in addition to little constraints from nuclear scattering and reactions. Each Skyrme parameterization is characterized by definite NM properties, such as symmetry energy and its density slope, incompressibility, and effective mass. The effective NN interactions in the form of Skyrme energy–density functionals successfully allowed the semi-microscopic self-consistent description of nuclear structure^28–32^, reactions^9,33–36^, and decays^37–41^, as well as nuclear matter properties^42–44^ and astrophysical phenomena^45,46^ investigations. It has been applied extensively in mean-field studies for several decades. Even so, some of the proposed parameterizations of the Skyrme energy density functional (EDF) could yield quite different values of the investigated quantity^3^, although they give similar agreement with the ground state properties of finite nuclei and with the NM saturation properties^47^. This takes place if the parameterization of the EDF is poorly obtained according to certain constrains, but it cannot be generalized to the different nuclear phenomena. So, a proper choice of the considered parameters is required in advance for a successful investigation of any phenomena or quantity in various nuclear topics, especially over wide ranges of density, spin, and temperature. The availability of proposed hundreds of parameter sets of the Skyrme EDF of the NN interaction allows systematic investigation of the impact of changing the various parameters characterizing its different terms on the physical results obtained in the mean field calculations. In the present study we investigate these influences on the proton and neutron density profiles of finite nuclei, based on twenty five Skyrme parameterizations that successfully predict the ground-state nuclear properties.

The paper is arranged as follows. After outlining the theoretical framework of the Skyrme effective NN interaction and its usage in the mean field calculations in Section "Theoretical framework", we present, describe and discuss the obtained density distributions based on the considered Skyrme forces, and the influences of the different force terms on them, in Section "Results and discussion". The summary and main conclusions are given in Section "Summary and conclusions".

## Theoretical framework

The self-consistent Hartree–Fock (HF) model based on Skyrme NN interaction is one of the semi-microscopic non-relativistic methods successfully used to find nuclear structure^48^. In this framework, we can find the total energy of a nuclear system by adding the kinetic and potential (Skyrme), parts to the Coulomb term, and considering pairing and shell contributions, in addition to a correction term that approximately eliminates the excitation energy of pseudo center-of-mass motion due to broken symmetries^48,49^,

1 $$E\left( {\rho_{p} ,\rho_{n} } \right) = \int {{\mathcal{H}}_{Sk} {\varvec{dr}} + \delta E_{ pairing + shell + correc} }.$$

In the standard form of the Skyrme-like effective NN interaction^50,51^, the nuclear energy density (${\mathcal{H}}_{Sk} ({\varvec{r}})$) reads

2 $${\mathcal{H}}_{Sk} \left( {\rho_{n,p} , \tau_{n,p} , \mathop{J}\limits^{\rightharpoonup} _{n,p} } \right) = {\mathcal{H}}_{K} \left( {\tau_{n,p} } \right) + {\mathcal{H}}_{0} + {\mathcal{H}}_{3} + {\mathcal{H}}_{eff} + {\mathcal{H}}_{fin} + {\mathcal{H}}_{so} + {\mathcal{H}}_{sg} + {\mathcal{H}}_{C} \left( {\rho_{p} } \right).$$

In this form, ${\mathcal{H}}_{K}$ represents the kinetic energy term given in terms of the kinetic energy density of protons ($\tau_{p}$) and neutrons ($\tau_{n}$) as,

3 $${\mathcal{H}}_{K} \left( {\varvec{r}} \right) = \frac{{\hbar^{2} }}{2m}\left[ {\tau_{p} \left( {\varvec{r}} \right) + \tau_{n} \left( {\varvec{r}} \right)} \right].$$

The central part of the Skyrme energy density functional given by Eq. (2) consists of the zero-range (${\mathcal{H}}_{0}$), density-dependent (${\mathcal{H}}_{3}$), effective-mass (${\mathcal{H}}_{eff}$), and finite-range (${\mathcal{H}}_{fin}$) contributions given respectively as,

4 $${\mathcal{H}}_{0} \left( {\varvec{r}} \right) = \frac{{t_{0} }}{2}\left[ {\left( {1 + \frac{1}{2}x_{0} } \right)\rho^{2} - \left( {x_{0} + \frac{1}{2}} \right)\left( {\rho_{p}^{2} + \rho_{n}^{2} } \right)} \right],$$

5 $${\mathcal{H}}_{3} \left( {\varvec{r}} \right) = \frac{1}{12}t_{3} \rho^{\sigma } \left[ {\left( {1 + \frac{1}{2}x_{3} } \right){\uprho }^{2} - \left( {x_{3} + \frac{1}{2}} \right)\left( {\rho_{p}^{2} + \rho_{n}^{2} } \right)} \right],$$

6 $${\mathcal{H}}_{eff} \left( {\varvec{r}} \right) = \frac{1}{4}\left[ {t_{1} \left( {1 + \frac{1}{2}x_{1} } \right) + t_{2} \left( {1 + \frac{1}{2}x_{2} } \right)} \right]\tau \rho + \frac{1}{4}\left[ {t_{2} \left( {x_{2} + \frac{1}{2}} \right) - t_{1} \left( {x_{1} + \frac{1}{2}} \right)} \right]\left( {\tau_{p} \rho_{p} + \tau_{n} \rho_{n} } \right),$$

and

7 $${\mathcal{H}}_{fin} \left( {\varvec{r}} \right) = \frac{1}{16}\left[ {3t_{1} \left( {1 + \frac{1}{2}x_{1} } \right) - t_{2} \left( {1 + \frac{1}{2}x_{2} } \right)} \right]\left( {\nabla \rho } \right)^{2} - \frac{1}{16}\left[ {3t_{1} \left( {x_{1} + \frac{1}{2}} \right) + t_{2} \left( {x_{2} + \frac{1}{2}} \right)} \right]\left( {\left( {\nabla \rho_{n} } \right)^{2} + \left( {\nabla \rho_{p} } \right)^{2} } \right).$$

$\rho_{i = p,n}$ and $\rho$ represent the protons, neutrons, and total local densities. *t*_*i* = 0,2,2,3_, *x*_*i*_ , and σ respectively denote the standard, exchange terms, and density dependence power parameters of the Skyrme effective interaction. Also, the spin–orbit (${\mathcal{H}}_{so}$) and the tensor coupling with spin and gradient (${\mathcal{H}}_{sg} )$ contributions of ${\mathcal{H}}_{Sk}$ are defined as

8 $${\mathcal{H}}_{so} \left( {\varvec{r}} \right) = \frac{{W_{0} }}{2}\left( {\mathop{J}\limits^{\rightharpoonup} \cdot \mathop{\nabla }\limits^{\rightharpoonup} \rho + \mathop \sum \limits_{i = p,n} \mathop{J}\limits^{\rightharpoonup} _{i} \cdot \mathop{\nabla }\limits^{\rightharpoonup} \rho_{i} } \right).$$

and

9 $${\mathcal{H}}_{sg} \left( {\varvec{r}} \right) = \frac{1}{16}\left[ {\left( {t_{1} - t_{2} } \right)\mathop \sum \limits_{i = p,n} \mathop{J}\limits^{\rightharpoonup2} _{i} - \left( {t_{1} x_{1} + t_{2} x_{2} } \right)\mathop{J}\limits^{\rightharpoonup2} } \right].$$

$W_{0}$ is the strength of the zero-range spin–orbit interaction, while $J_{i = p,n}$ and $J$ represent the protons, neutrons, and total spin–orbit densities. The Skyrme parameters are usually derived to reproduce the nuclear structure and nuclear matter properties, as well as different nuclear phenomena.

The Coulomb energy contribution can be obtained in terms of the proton density distribution as

10 $$H_{Coul} = H_{C}^{dir} + H_{C}^{exch} = \frac{{e^{2} }}{2}\rho_{p} \left( {\mathop{r}\limits^{\rightharpoonup} } \right)\int {\frac{{\rho_{p} (\mathop{r}\limits^{\rightharpoonup\prime } )}}{{\left| {\mathop{r}\limits^{\rightharpoonup} - \mathop{r}\limits^{\rightharpoonup\prime } } \right|}}d\mathop{r}\limits^{\rightharpoonup\prime } } - \frac{{3e^{2} }}{4}\left( {\frac{3}{\pi }} \right)^{\frac{1}{3}} \left( {\rho_{p} \left( {\mathop{r}\limits^{\rightharpoonup} } \right)} \right)^{\frac{4}{3}} .$$

The two terms in this equation represent the direct ($H_{C}^{dir}$) and the exchange ($H_{C}^{exch}$) contributions of the Coulomb energy. The exchange part is expressed based on in the Slater approximation^52^.

In the self-consistent HF calculations based on Skyrme NN interaction, the density distributions ($\rho_{i = p,n}$) of protons and neutrons, the kinetic ($\tau_{i}$), and the spin–orbit ($\mathop{J}\limits^{\rightharpoonup} _{i}$) densities can be obtained using the single-particle wave functions (${\upvarphi }^{l}$($\mathop{r}\limits^{\rightharpoonup} ,\sigma$)) and the corresponding occupation numbers ($n_{{\beta_{i} }}$), with orbital (*l*) and spin ($\sigma$) quantum numbers ($\beta = (l,\sigma )$), by the sum over the involved single-particle states^50,51,53^, as

11 $$\rho_{i = p\left( n \right)} \left( {\mathop{r}\limits^{\rightharpoonup} } \right) = \mathop \sum \limits_{{\beta_{i} }} \left| {{\upvarphi }_{i}^{l} \left( {\mathop{r}\limits^{\rightharpoonup} ,\sigma } \right)} \right|^{2} n_{{\beta_{i} }} ,$$

12 $$\tau_{i} \left( {\mathop{r}\limits^{\rightharpoonup} } \right) = \mathop \sum \limits_{{\beta_{i} }} \left| {\nabla {\upvarphi }_{i}^{l} \left( {\mathop{r}\limits^{\rightharpoonup} ,\sigma } \right)} \right|^{2} n_{{\beta_{i} }} ,$$

and

13 $$\mathop{J}\limits^{\rightharpoonup} _{i} \left( {\mathop{r}\limits^{\rightharpoonup} } \right) = \mathop \sum \limits_{{\beta_{i} \left( {l,\sigma } \right),\sigma^{\prime } }} {\upvarphi }_{i}^{l*} \left( {\mathop{r}\limits^{\rightharpoonup} ,\sigma^{\prime } } \right)\nabla {{\upvarphi }}_{i}^{l} \left( {\mathop{r}\limits^{\rightharpoonup} ,\sigma } \right) \times \langle\sigma^{\prime } {|}\mathop{\sigma }\limits^{\rightharpoonup} {|}\sigma\rangle n_{{\beta_{i} }} .$$

For spherical nuclei of stationary ground-state, the single-particle wave function can be represented as a combination of radial part $R_{\beta }$ and separate spinor spherical harmonics $Y_{{j_{\beta } \ell_{\beta } m_{\beta } }}$^53^,

14 $${\upvarphi }_{\beta } \left( {\mathop{r}\limits^{\rightharpoonup} } \right) = \frac{{R_{\beta } \left( r \right)}}{r}Y_{{j_{\beta } \ell_{\beta } m_{\beta } }} \left( {\theta ,\phi } \right).$$

While $m_{\beta }$ does not influence the radial properties, different values of the quantum number $j_{\beta }$ and $\ell_{\beta }$ remain multiple, where the harmonic-oscillator shell model is frequently used to describe the shell order of the single-particle states^53^. Consequently, the matter, kinetic, and spin–orbit densities can be radially represented. The total density is then the sum of the associated local proton and neutron densities. The pairing correction can be considered in the performed calculations by the Bardeen-Cooper-Schriffer (BCS) method, with a constant gap approximation^53^, which will be considered in the present work, or by constant or density-dependent zero-range force^50^. The pairing energy functional can be schematically represented in terms of constant paring matrix elements ($G_{i}$) as

$$H_{Pair} = - \mathop \sum \limits_{i = p,n} G_{i} \left[ {\mathop \sum \limits_{\beta \in i} \sqrt {n_{\beta } \left( {1 - n_{\beta } } \right)} } \right]^{2} .$$

The BCS occupation weights ($n_{\beta }$) are defined in terms of Fermi energy ($\in_{Fi}$) and pairing gap ($\Delta_{i}$) as,

$$n_{\beta } = \frac{1}{2}\left[ {1 - \frac{{ \in_{\beta - } \in_{Fi} }}{{\sqrt {\left( { \in_{\beta - } \in_{Fi} } \right)^{2} + \Delta_{i}^{2} } }}} \right].$$

Considering constant force treatment of pairing, $\in_{Fi}$ and $\Delta_{i}$ are simultaneously determined by the particle number ($N_{i} = \sum\nolimits_{\beta \in i} {n_{\beta } }$) condition and the gap equation ($\Delta_{i} /G_{i} = \sum\nolimits_{\beta \in i} {\sqrt {n_{\beta } \left[ {1 - n_{\beta } } \right]} }$). The pairing gap might be also parameterized by a constant gap approximation^48,53^. An averaged pairing gap of ${\tilde{\Delta }} = \frac{11.2}{{\sqrt A }}{\text{ MeV}}$ is acceptable to give good agreement along the nuclear chart. The coupled HF and pairing equations are then solved through simultaneous iteration of ${\upvarphi }_{\beta }$ and the occupation weights.

To obtain the charge density from the HF + BCS calculation, we have to take into consideration the intrinsic electromagnetic structure of nucleons. This can be performed by folding the obtained proton and neutron densities with the intrinsic charge density of the nucleons in Fourier space, through their corresponding form factors,

$$F_{i} \left( k \right) = 4\pi \int_{0}^{\infty } {dr r^{2} j_{0} \left( {kr} \right)\rho_{i} \left( r \right).}$$

*j*_0_ denotes the zero-order spherical Bessel function. Then, the charge form factor is given as^53^,

$$F_{C} \left( k \right) = \mathop \sum \limits_{i} \left[ {F_{i} \left( k \right)G_{Ei} \left( k \right) + F_{ls,i} \left( k \right)G_{M} \left( k \right)} \right]\exp \left( {\frac{{\hbar^{2} k^{2} }}{{8P_{cm}^{2} }}} \right).$$

Here, *G*_*Ei*_, *G*_*M*_, and $F_{ls,i} \left( k \right)$ respectively denote the electric and magnetic form factors of nucleons, and the form factor of the spin–orbit current ∇***J***. The exponential factor considers unfolding of the spurious center of mass vibrations in the harmonic approximations. The intrinsic form factors of nucleons are obtained from electron scattering data on protons and deuterons. Finally, the charge density is obtained using the inverse Fourier–Bessel transform of the charge form factor. The nucleon densities are obtained on a finite radial grid, using the computed form factor that stored in the form of reciprocal lattice *F*(*k*_*j* = 1 − *Nr*_), where *k*_*j*_ = (*j* − 1) π/*Δ*_*r*_* N*_*r*_. The defined grid (*k*_*j* = 1 − Nr_) in coordinate space is usually finer than that in momentum space, because the finite difference formulas for kinetic energy become inferior to a Fourier representation. The intermediate values of *F*(*k*) are computed by Fourier–Bessel interpolation, in terms of the length of the coordinate grid (*Δ*_*r*_* N*_*r*_). For sufficient precision in energy and radius with a reasonable grid size, the number of the radial grid points (*N*_*r*_) and grid spacing (*Δ*_*r*_) increase with increasing the number of nucleons.

The proton, neutron, and charge root mean square radii are then obtained as,

15 $$R_{p,n,C}^{rms} = \left\langle {R_{p,n,C}^{2} } \right\rangle^{1/2} = \left( {\frac{{\int {r^{2} \rho_{p,n,C} \left( {\mathop{r}\limits^{\rightharpoonup} } \right)d\mathop{r}\limits^{\rightharpoonup} } }}{{\int {\rho_{p,n,C} \left( {\mathop{r}\limits^{\rightharpoonup} } \right)d\mathop{r}\limits^{\rightharpoonup} } }}} \right)^{1/2} .$$

For normalized proton (neutron) density distributions, the denominator becomes *Z* (*N*) of the nucleus. Also, we can calculate the neutron-skin thickness ($\Delta_{np}$) that represents the extension of the neutron density with respect to the proton density as,

16 $$\Delta_{np} \left( {A,Z,N} \right) = \langle R_{n}^{2}\rangle^{1/2} - \langle R_{p}^{2}\rangle^{1/2} .$$

For a practical use in the nuclear structure and reaction studies, one would express the proton (neutron) density of spherical nuclei in terms of the corresponding half-density radius ($R_{p\left( n \right)}$) and diffuseness ($a_{n\left( p \right)}$) in the two-parameter Fermi (2pF) form,

17 $$\rho_{p\left( n \right)} \left( r \right) = \rho_{{0p\left( {0n} \right)}} \left( {1 + e^{{(r - R_{p(n)} )/a_{p(n)} }} } \right)^{ - 1} ,$$

where *r* denotes the distance measured from the center of the nucleus. We can then fit the density distributions evaluated from the self-consistent Skyrme HF + BCS calculations in the 2pF form. To obtain accurate values of the half-density radius and diffuseness, far from the inner fluctuations of the numerically calculated density, one can fit the obtained density to the function $r^{2} \rho$ instead of fitting to $\rho$ itself. The obtained 2pF form of the density is then normalized to Z (N) of the nucleus to find the value of the $\rho_{{0p\left( {0n} \right)}}$ parameter.

Based on the Skyrme EDF, we can express the energy per nucleon for infinite asymmetric nuclear matter (ANM) as $E_{A} = {\mathcal{H}}_{Sk} \left( \rho \right)/\rho$^54^. Based on the expansion of $E_{A}$ in terms of *ρ* and the proton fraction *η* = *Z*/*A*, we can also derive the symmetry energy *E*_*sym*_ (ρ) that scale the isospin dependence of the NN interaction as $E_{sym} \left( \rho \right) = \left( {1/8} \right)\left. {\partial^{2} E_{A} \left( {\rho ,\eta } \right)/\partial \eta^{2} } \right|_{\eta = 1/2} .$ The main characteristic quantities for the Equation of State (EOS) of ANM that control its behavior are the coefficients of the symmetry energy (*a*_*sym*_ = *E*_*sym*_(*ρ*_0_)) and its density slope $L = 3\rho_{0} \left. {\partial E_{sym} \left( \rho \right)/\partial \rho } \right|_{{\rho_{0} }}$, and the incompressibility coefficient $K_{0} = 9\rho \left. {\partial^{2} E_{A} \left( \rho \right)/\partial \rho^{2} } \right|_{{\rho_{0} }}$, which are defined at normal saturation density (*ρ*_0_). Also, the effective nucleon mass (m*) relative to the free nucleon mass (*m*) can be derived in terms of the single particle energy (*ε*_*i*_) and momentum (p) as m*/m = *(m/p) dε*_*N*_*/dp*. The isoscalar (IS) and isovector (IV) effective masses, which are respectively related to the symmetric and asymmetric properties of nuclear matter, are given as

18 $$\left( {\frac{{m^{*} }}{m}} \right)_{{i\left( {{\text{IS}}} \right)}}^{ - 1} = 1 + \frac{{m_{i} }}{{8\hbar^{2} }}\left[ {3t_{1} + t_{2} \left( {5 + 4x_{2} } \right)} \right]\rho_{i}$$

and

19 $$\left( {\frac{{m^{*} }}{m}} \right)_{{i\left( {{\text{IV}}} \right)}}^{ - 1} = 1 + \frac{{m_{i} }}{{4\hbar^{2} }}\left[ {t_{1} \left( {2 + x_{1} } \right) + t_{2} \left( {2 + x_{2} } \right)} \right]\rho_{i} .$$

Each Skyrme EDF has its own parameters and values of the characteristic nuclear matter properties, which in turn affect the different isospin-asymmetry and density-dependent properties of finite nuclei and their reactions, when evaluated based on its parameterization^9,44,49,54–56^.

## Results and discussion

Towards our aim, we consider many Skyrme NN interactions which cover the widest possible ranges of both the different Skyrme parameters (*t*_*i*=0,2,2,3_, *x*_*i*_, σ, and *W*_*0*_) and the saturation properties (*J*, *L*, *K*^0^, and m*/*m*) of the corresponding EOS of asymmetric nuclear matter. Specifically, twenty five Skyrme parameterizations have been chosen, namely the SkSC1,3,5,6,10,14^57–59^, SkM1^58^, RATP^60^, Es^61^, SkT3,5^62^, KDE0v^63^, KDE0v1^63^, KDEX^64^, SLy4^50^, SkI2^65^, SII^66^, Skxs20,25^67^, SkI5^65^, SK272^68^, Ska35s25^54^, SGOI^69^, and BSk19,21^70^ parameterizations. As each set of parameters is complementary to its corresponding force, the effect of any term or of its corresponding parameters is needed to be analyzed simultaneously through the different parameterizations, and not to change its value individually. The mentioned forces will be used to find the density distributions of the spherical ^30^Ne, ^32^Mg, ^36^S, ^38^Ar, ^40^Ca, ^54^Fe, ^60^Ni, ^66^Zn, ^86^Kr, ^92^Mo, ^100,132^Sn, ^142^Nd, ^152^Yb, ^186,208^Pb, ^216^Th, and ^218^ U nuclei, which cover wide ranges of charge number 10 (Ne) ≤ Z ≤ 92 (U), neutron number 20 (^30^Ne, ^32^Mg, ^36^S, ^38^Ar, ^40^Ca) ≤ N ≤ 126 (^208^ Pb, ^216^Th, ^218^ U), and isospin asymmetry 0 (^40^Ca, ^100^Sn) ≤ I = (N-Z) ≤ 0.333 (^30^Ne). The present HF + BCS calculations^53^ based on the different considered forces show that among these isotopes, only ^40^Ca^71,72^, ^60^Ni^73^, and ^100^Sn exhibit a thin proton-skin, of a thickness less than 0.1 fm. The ^54^Fe^74^ and ^152^Yb isotopes show almost equal neutron and proton radii. The isotopes ^38^Ar, ^66^Zn, ^92^Mo, ^186^Pb, and ^218^ U show thin neutron-skin thickness less than 0.1 fm. The remaining ^30^Ne, ^32^Mg, ^36^S, ^86^Kr, ^132^Sn^73^, ^142^Nd, ^208^Pb^73^, and ^216^Th isotopes show a clear thick neutron-skin thickness larger than 0.1 fm.

### The central part of the Skyrme EDF

**Figure 1:**
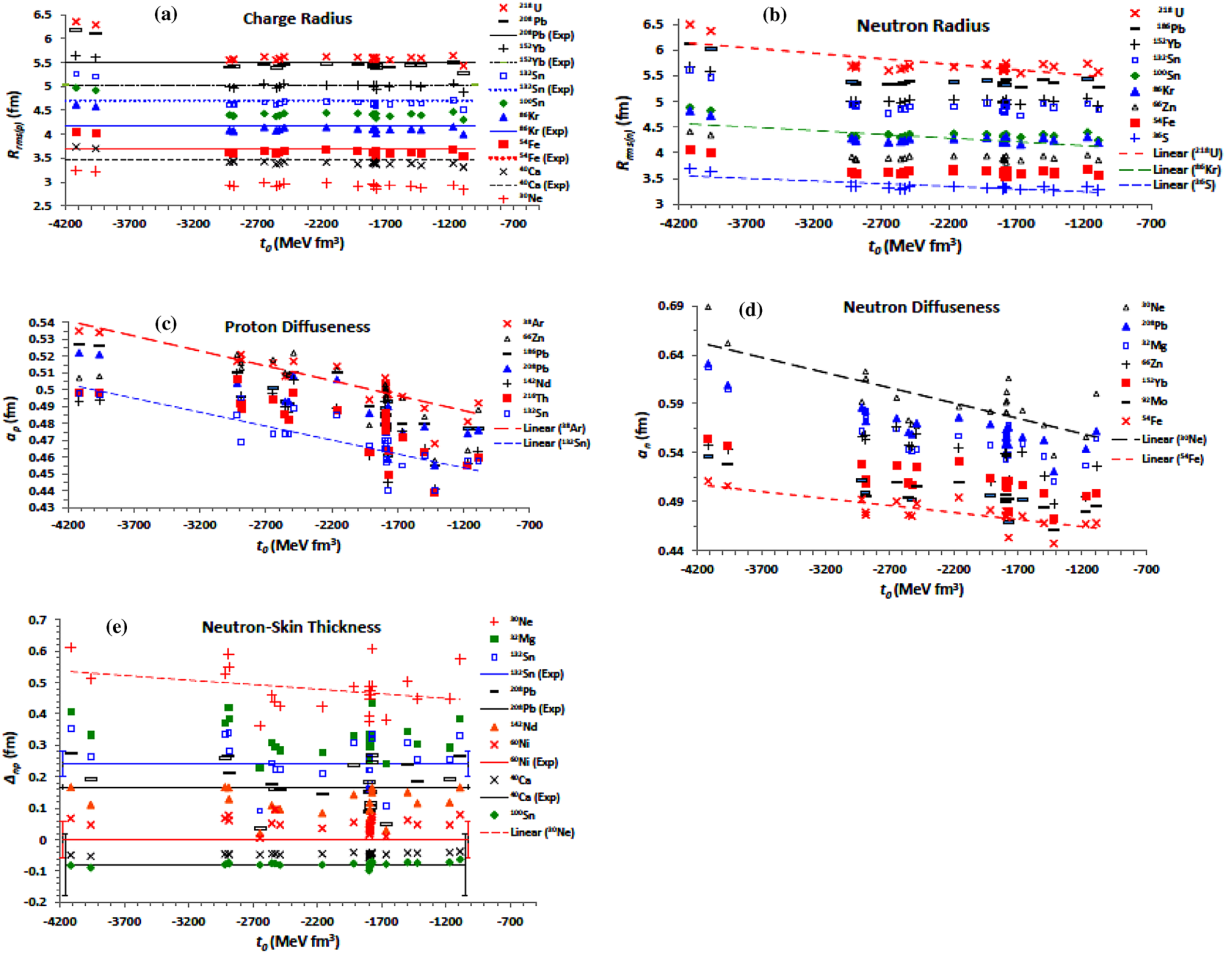
The rms radii of the calculated (**a**) charge and (**b**) neutron density distributions, the corresponding (**c**) proton and (**d**) diffuseness parameters, and the related (**e**) neutron-skin thickness of the nuclei mentioned in the different figure panels, as functions of the *t*_0_ parameter of the twenty-five Skyrme forces mentioned in the text. The sold and dotted straight lines in panels (**a**) and (**e**) denote experimental data of the rms charge radii^75,76^ and of the neutron-skin thickness, respectively, for the mentioned isotopes. To guide the eyes, the dashed lines represent trend lines for the corresponding data.

**Figure 2:**
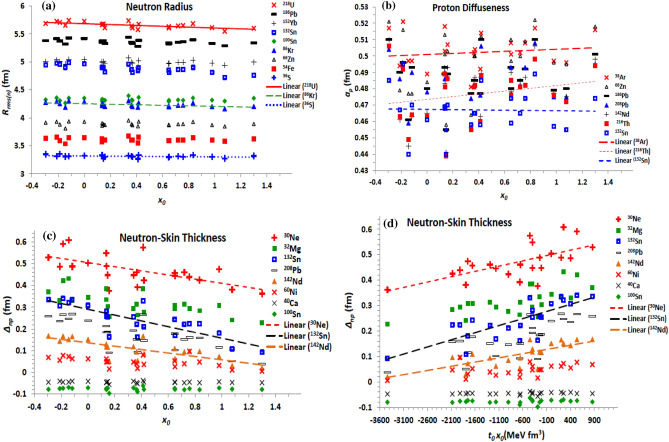
Same as Fig. 1b, c and e, respectively, but the (**a**) neutron rms radius, (**b**) proton diffuseness, and (**c**) neutron-skin thickness are displayed against the exchange parameter (*x*_0_) of the zero-range term of the Skyrme effective interaction. (**d**) The neutron-skin thickness is displayed against the combination *t*_0_*x*_0_ of the zero-range term.

We start with the central part of the effective EDF of the NN interaction which contains the zero-range ${\mathcal{H}}_{0}$($t_{0}$,$x_{0}$), density-dependent ${\mathcal{H}}_{3} \left( {\sigma ,t_{3} , x_{3} } \right)$, effective-mass ${\mathcal{H}}_{eff} (t_{1,2} , x_{1,2}$), and the finite-range $(t_{1,2} , x_{1,2} )$ contributions. Figure 1 shows the root mean square (rms) radii of the charge (*R*_*rms*(*p*)_) and neutron (*R*_*rms(n)*_) density distributions, and the corresponding diffuseness parameter (*a*_*p(n)*_), and the neutron-skin thickness (*∆*_*np*_), as functions of *t*_0_, for the selected isotopes presented on panels (a)–(e) respectively. The presented isotopes in each panel are selected to provide a good resolution of the displayed data. The experimental data of the rms charge radii^75,76^ of the ^208^Pb, ^152^Yb, ^132^Sn, ^86^Kr, ^54^Fe and ^40^Ca isotopes, and that of the neutron-skin thickness^71–73,77^ of the ^132^Sn, ^208^Pb, ^60^Ni, and ^40^Ca isotopes are displayed by the solid and dotted straight lines in Fig. 1a and e, respectively. The dashed straight lines in Fig. 1b–e represent trend lines for the corresponding data, to guide the eyes. The trend lines are plotted according to their better coefficient of determination R^2^. As clearly seen in Fig. 1, the most effect of the value of *t*_*0*_ coefficient related to the zero-range part of the central EDF is reflected in the diffuseness values of both proton (Fig. 1c) and neutron (Fig. 1d) density distributions, which strongly decrease upon increasing *t*_*0*_. With less rate, both the rms charge (Fig. 1a) and neutron (Fig. 1b) radii, and the neutron-skin thickness (Fig. 1e) also tend to decrease with increasing *t*_*0*_. In the next figures, we will exclude the extreme large values of *R*_*rms(p)*_ and *a*_*p(n)*_ corresponding to the extreme values of *t*_0_ =  − 4115 MeV fm^3^ (BSk19) and *t*_0_ =  − 3961 MeV fm^3^ (BSk21) so as not to affect our conclusions regarding the other coefficients. The effect of the exchange parameter (*x*_0_) of the zero-range term of the EDF is displayed in Fig. 2. Although the − 0.3 ≤ *x*_0_ ≤ 1.3 coefficient has smaller values than − 4115 ≤ *t*_0_ (MeV fm^3^) ≤  − 1089, they are equally affecting as they are multiplied together (*t*_0_ *x*_0_) in the zero-range term. While the neutron radius slightly decrease with increasing *x*_0_ (Fig. 2a), the calculations show that the charge radius tends to be constant. On the other hand, the proton diffuseness shows weak decreasing behaviour with *x*_0_ in Fig. 2b, while the calculations indicate that neutron diffuseness remains almost constant with increasing *x*_0_. The overall effect of changing the proton and neutron density profiles with *x*_0_ appears as increasing behaviour of the neutron-skin thickness with *x*_0_, as seen in Fig. 2c. As the neutron-skin thickness decreases (increases) with increasing *t*_0_ (*x*_0_), Fig. 1d shows the net behaviour of *∆*_*np*_ with increasing the combination *t*_0_*x*_0_ of the zero-range term. Figure 1d shows *∆*_*np*_ increases upon increasing the *t*_0_*x*_0_ coefficient.

**Figure 3:**
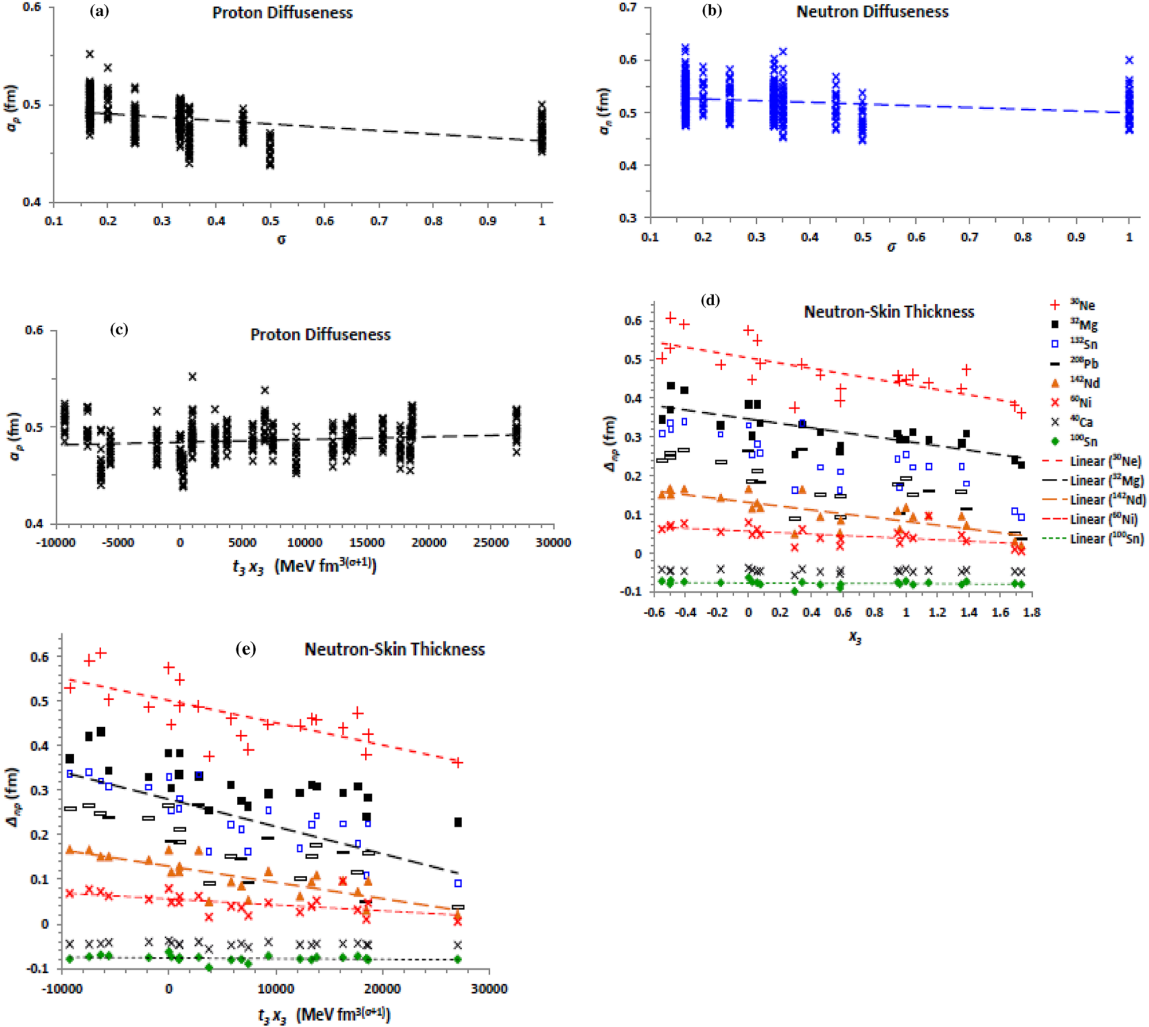
Same as Fig. 1c, d and e, but the (**a**) proton and (**b**) neutron diffuseness are displayed against the power σ of the density dependence term. (**c**) The proton diffuseness is displayed versus the *t*_3_ parameter, and (**d**) The neutron-skin thickness is displayed versus the exchange parameter *x*_3_, of the density dependence term of the Skyrme effective interaction.

Figure 3 displays the dependence of the density distributions on the parameters of the density-dependent term ${\mathcal{H}}_{3} \left( {\sigma ,t_{3} , x_{3} } \right)$ of the central part of the EDF. The proton and neutron diffuseness parameters for all nuclei under study are presented in Fig. 3a and b, respectively, as functions of the power σ of the density dependence. The proton diffuseness is plotted as a function of the combination *t*_3_*x*_3_ in Fig. 3c. The behavior of the neutron-skin thickness against the exchange density-dependence parameter (*x*_3_) and against the combination *t*_3_*x*_3_, are shown in Fig. 3d and e, respectively, for a few isotopes. The most significant effect for the density dependence term is obtained as a decreasing behaviour of both the proton (Fig. 3a) and neutron (Fig. 3b diffuseness parameters with the power σ, and their increasing behaviour with *t*_3_. Increasing the exchange parameter *x*_*3*_ increases *a*_*p*_ (decrease *a*_*n*_) slightly. The two densities slightly increase upon increasing the combination *t*_*3*_*x*_*3*_, as clearly seen in panel 3c for *a*_*p*_. Moreover, Fig. 3d shows that the neutron-skin thickness tends to decrease with increasing *x*_3_. The effect of changing *x*_3_, which governs the surface-symmetry properties^62^, on the diffuseness and the neutron-skin thickness generally increases with increasing the isospin asymmetry of the nucleus, as seen in Fig. 3d for *∆*_*np*_. The calculations based on the different considered forces show that increasing the power σ and the *t*_3_ parameter slightly increases *∆*_*np*_. The proton and neutron radii did not significantly affected with the change of the three σ, *t*_3_, and *x*_3_ parameters. Expectedly, the effect of the variation of the power σ exceeds that of linear *t*_3_ and *x*_3_ parameters of the density dependence term, and then it leads the effect of the density dependence term on the density distribution.

**Figure 4:**
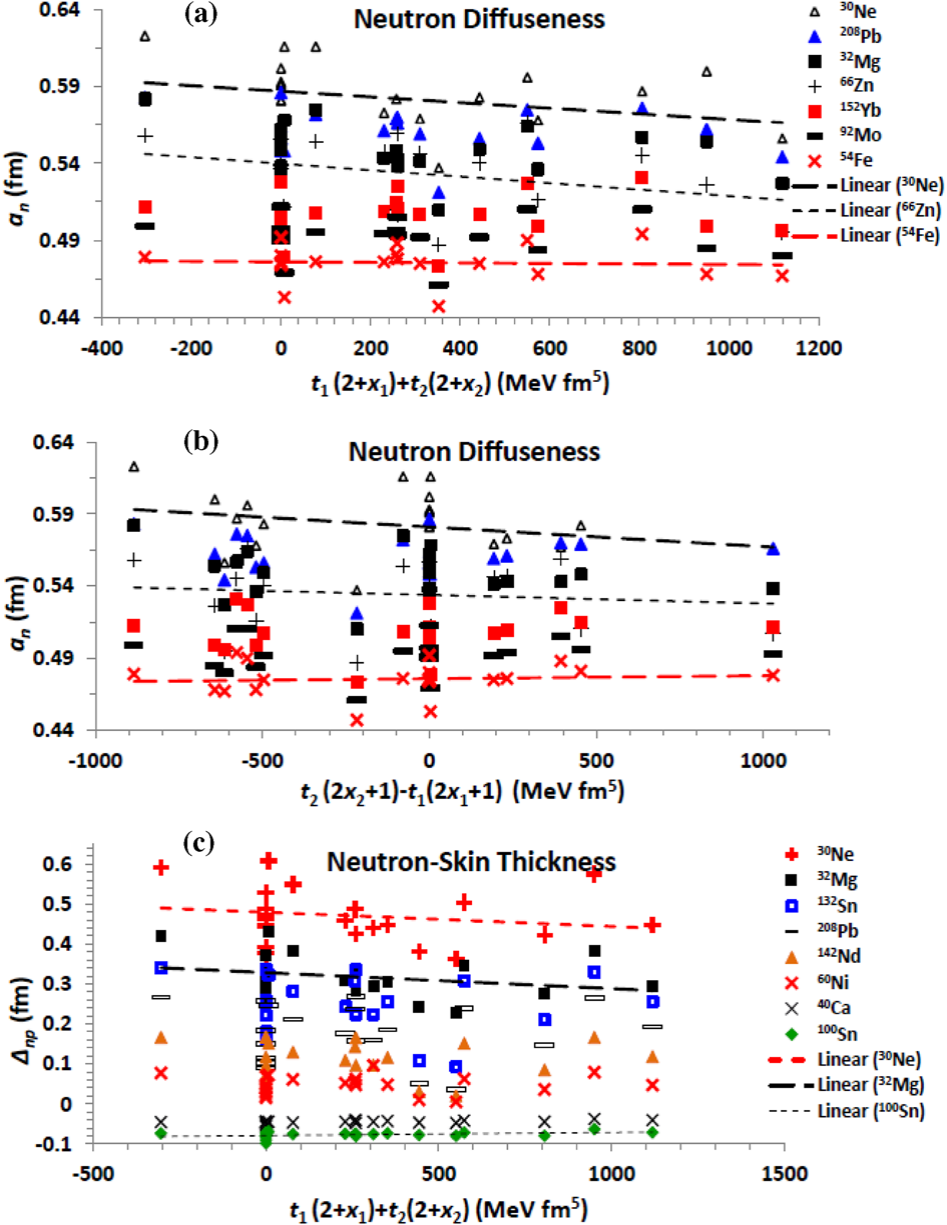
Same as Fig. 1d and e, but the neutron diffuseness is displayed against the combinations (**a**) *H*_*eff1*_ = *t*_1_(2 + *x*_1_) + *t*_2_(2 + *x*_2_) and (b) *H*_*eff2*_ = *t*_2_(2*x*_2_ + 1)-*t*_1_(2*x*_1_ + 1), of the effective mass term of the Skyrme effective interaction, and (c) The neutron-skin thickness is displayed versus *H*_*eff1*_ = *t*_1_(2 + *x*_1_) + *t*_2_(2 + *x*_2_).

**Figure 5:**
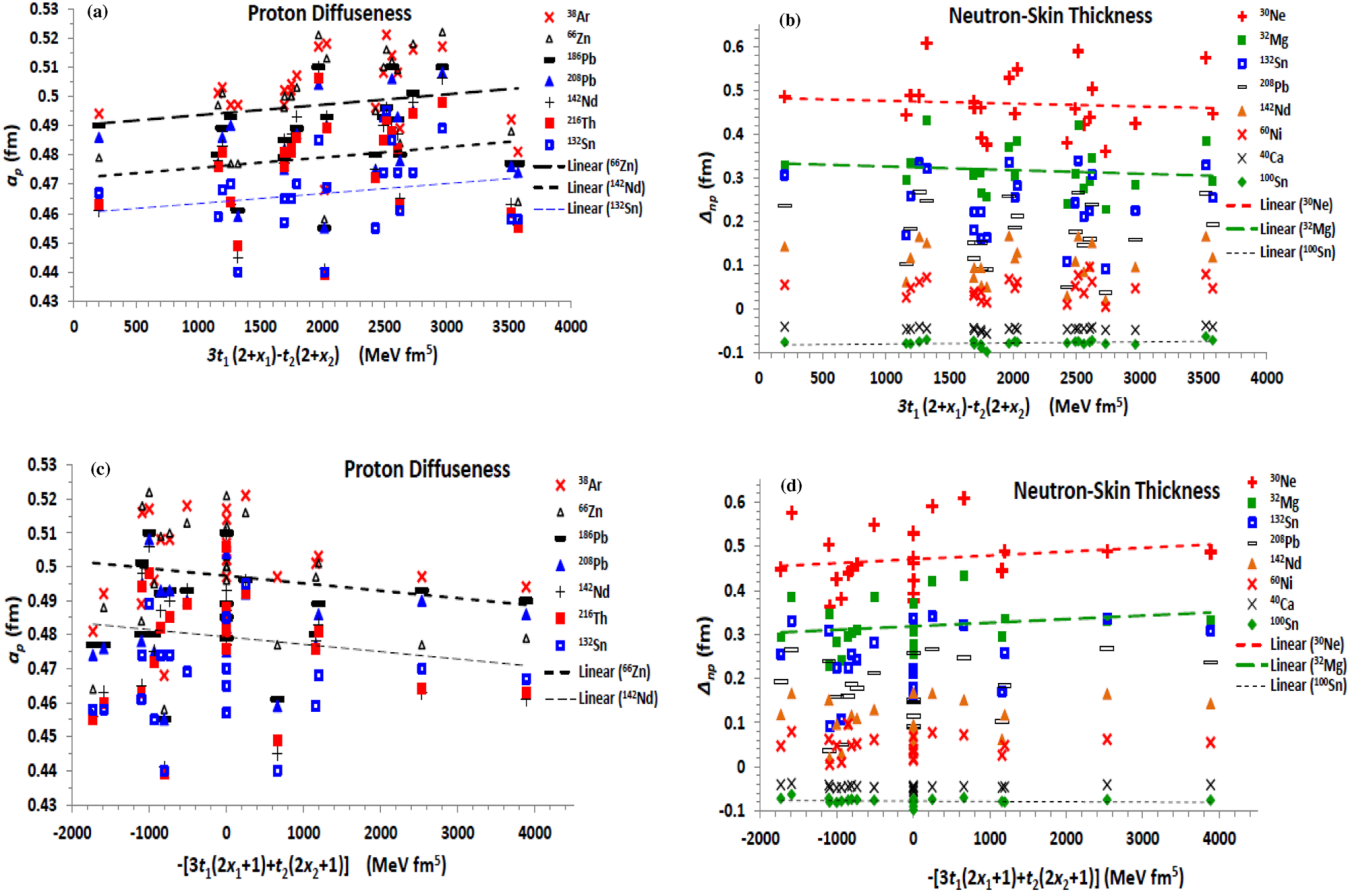
Same as Fig. 1c and e but the proton diffuseness and the neutron-skin thickness are displayed against the combinations (**a**) and (**b**) *H*_*fin1*_ = 3*t*_1_(2 + *x*_1_) − *t*_2_(2 + *x*_2_), (**c**) and (**d**) *H*_*fin2*_ =  − [3*t*_1_(2*x*_1_ + 1) + *t*_2_(2*x*_2_ + 1)] of the finite-range term of the Skyrme effective interaction.

The effective mass (${\mathcal{H}}_{eff}$) and finite-range (${\mathcal{H}}_{fin}$) non-local terms of the effective Skyrme interaction, as well as its J^2^ tensor term, are governed by the four *t*_1,2_ (MeV fm^5^) and *x*_1,2_ characteristic parameters. According to the performed calculations based on the considered parameterizations of the Skyrme EDF, the neutron and proton diffuseness generally decrease upon increasing both *t*_2_ and *x*_2_ parameters. *t*_1_ and *x*_1_ decrease also *a*_*n*_ but they slightly increase *a*_*p*_. The effect of changing the standard *t*_1,2_ parameters on both the density distributions is mostly more effective than that of the exchange *x*_1,2_ parameters. The proton and neutron radii show overall fixed trend with increasing the *t*_1,2_ and *x*_1,2_ coupling parameters of the non-local terms of the Skyrme interaction. The influences of the combinations *H*_*eff1*_ = *t*_1_(2 + *x*_1_) + *t*_2_(2 + *x*_2_) and *H*_*eff2*_ = *t*_2_(2*x*_2_ + 1) − *t*_1_(2*x*_1_ + 1), appearing in Eq. (6) of the effective mass term, on the neutron diffuseness are displayed in Fig. 4a and b, respectively. The effect of the combination *H*_*eff1*_ on the neutron-skin thickness is presented in Fig. 4c. As shown in Fig. 4a and b, increasing the *H*_*eff1*_ and *H*_*eff2*_ coefficients decreases the neutron diffuseness. The same behaviour is obtained for the proton diffuseness, but it decreases slightly with *H*_*eff2*_. The neutron-skin thickness also decreases slightly with increasing *H*_*eff1*_ (Fig. 4c) and *H*_*eff2*_. Thus, both the neutron and proton diffuseness, and the neutron-skin thickness tend to decrease with increasing the strength of the non-local effective mass term of the effective interaction. Figure 5 shows the effect of changing the explicit combinations *H*_*fin1*_ = 3*t*_1_(2 + *x*_1_) − *t*_2_(2 + *x*_2_) and *H*_*fin2*_ =  − [3*t*_1_(2*x*_1_ + 1) + *t*_2_(2*x*_2_ + 1)], appearing in the finite-range term given by Eq. (7), on the diffuseness of proton density, Fig. 5a and c, and on the neutron-skin thickness, Fig. 5b and d. According to the present results, and as seen in Fig. 5a and b, while increasing the first coefficient (*H*_*fin1*_) increases *a*_*p*_, it slightly diminishes both *a*_*n*_ and *∆*_*np*_. Increasing the value of the combination *H*_*fin2*_ of the second part of ${\mathcal{H}}_{fin}$ shows almost stronger opposite effect against increasing *H*_*fin1*_, where it decreases *a*_*p*_ (Fig. 5c), but it increases both *a*_*n*_ and *∆*_*np*_ (Fig. 5d). Thus, increasing the finite-range contribution of EDF of the effective interaction is expected to increases slightly both the diffuseness of neutrons and the neutron-skin thickness, keeping the proton diffuseness almost unchanged. This is because the effect of increasing *a*_*n*_ and *∆*_*np*_ with *H*_*fin2*_ (Fig. 5d) is more effective than their slight decreasing with *H*_*fin1*_ (Fig. 5b), but both *H*_*fin1*_ and *H*_*fin2*_ show nearly equal opposite effects on *a*_*p*_, as indicated in Fig. 5a and c.

### The spin–orbit and tensor parts of the Skyrme EDF

**Figure 6:**
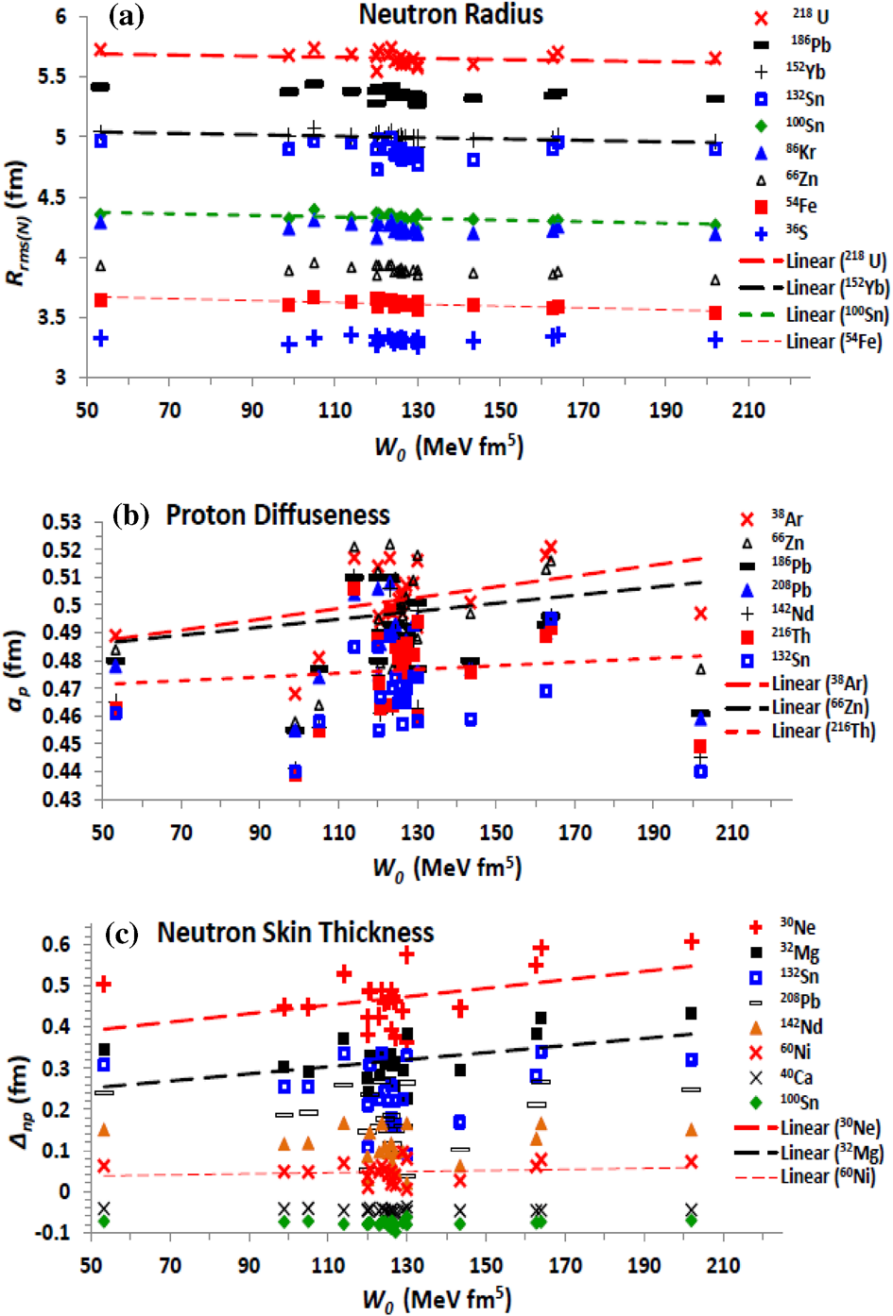
Same as Fig. 1b, c and e, but (**a**) the neutron rms radius, (**b**) proton diffuseness, and (**c**) the neutron-skin thickness are displayed versus the strength (*W*_0_) of spin–orbit term of the Skyrme effective interaction.

The strength (*W*_0_) of spin–orbit force given by Eq. (8) controls its impact on the related nuclear properties and interactions. For instance, it was indicated that the two-body spin–orbit contribution of the NN interaction slightly increase the absorption part of the optical potential in the interior region, affecting the angular distribution and analysing power of neutron elastic scattering by doubly closed-shell nuclei^34^. Figure 6 displays the dependence of the density distribution on *W*_0_. The present results show that increasing the spin–orbit strength slightly increases both the charge and neutron (Fig. 6a) rms radii, but increases the corresponding proton (Fig. 6b) and neutron diffuseness, and the related neutron-skin thickness (Fig. 6c). As shown in Fig. 6, the effect of changing the spin–orbit strength is stronger for the nuclei having larger isospin-asymmetry coefficient such as ^30^Ne, ^32^Mg, ^132^Sn, and ^208^Pb, than its effect on those having smaller isospin-asymmetry such as ^60^Ni and ^54^Fe and on the ^40^Ca and ^100^Sn spin-saturated nuclei. Increasing the binding of the nucleons with increasing the spin–orbit force is the reason why the proton and neutron radii slightly decrease with increasing the spin–orbit strength. Indeed, the spin–orbit force increases the binding of the nucleons in heavy nuclei, which occupy high orbits with large orbital angular momentum, than that of nucleons occupying lower orbits in light nuclei. This is one of the reasons why the diffuseness decreases for heavy nuclei. This also decreases the neutron-skin thickness of heavy nuclei relative to that of light nuclei of the same isospin-asymmetry, where the increase of orbital angular momenta of large number of neutrons in the higher orbits of heavy nuclei becomes more than it for the smaller number of protons occupying less number of orbits with less orbital angular momenta. Consequently, the neutrons become more bound in the heavy nuclei relative to the light nuclei of the same isospin asymmetry, while the binding of protons remains almost the same. This reduces the expected increase of the neutron radius due to increasing its number, with respect to the expected increase of the proton radius, which appears as a decrease the neutron-skin thickness. The maximum effect of increasing the spin–orbit force appears then for the light nuclei having large isospin asymmetry.

**Figure 7:**
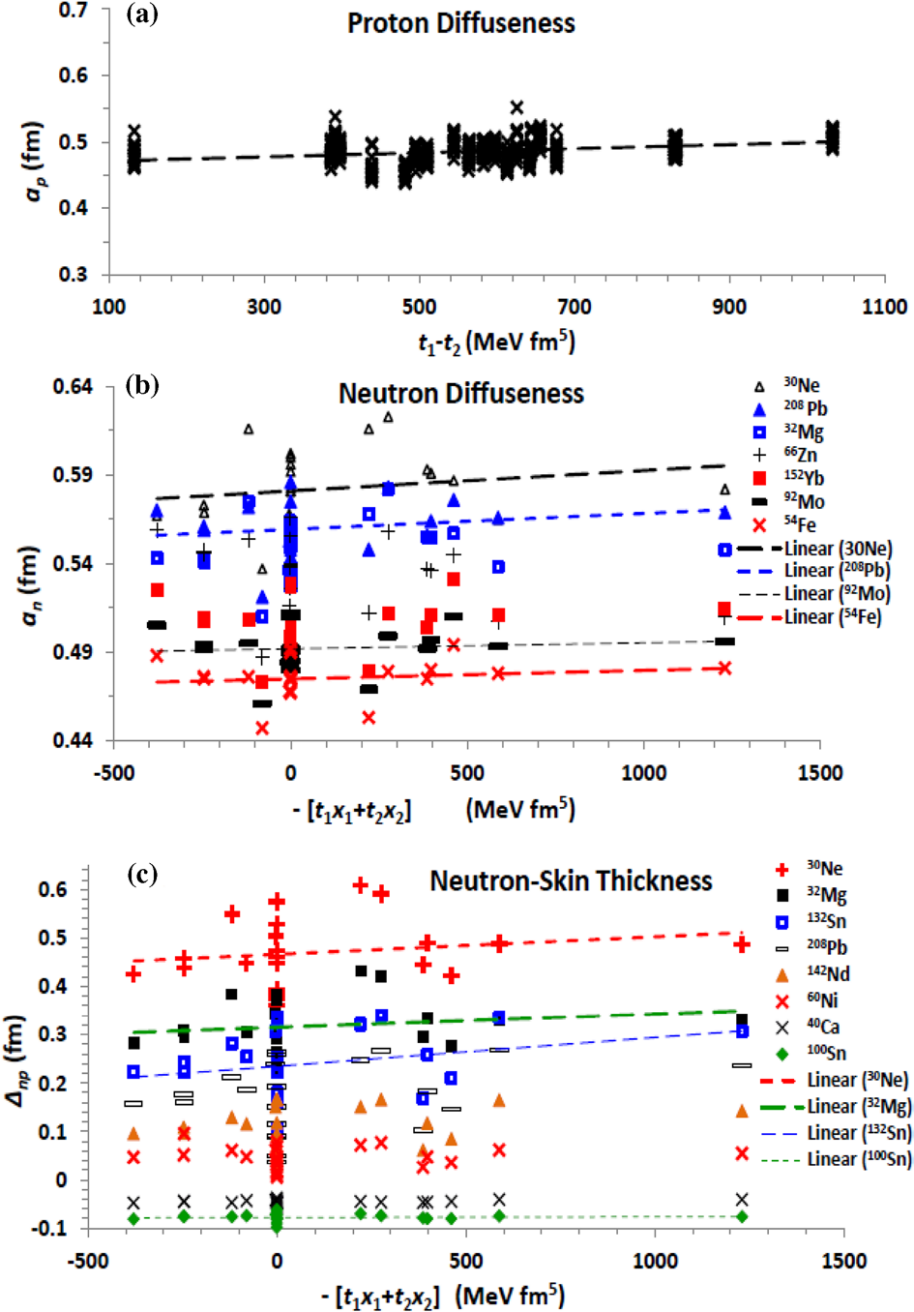
(**a**) The proton diffuseness against the combination (*t*_1_ − *t*_2_) of the J^2^ tensor term of the Skyrme effective interaction, for all investigated isotopes, and the (**b**) neutron diffuseness and (**c**) neutron-skin thickness displayed versus the combination *H*_*sg2*_ =  − (*t*_1_ *x*_1_ + *t*_2_ *x*_2_) of the same term, for the isotopes mentioned in the panels.

Now we come to the *J*^*2*^ tensor term (${\mathcal{H}}_{sg}$), which arises from both the zero-range central and tensor forces^78^. This term is governed by the difference combination *H*_*sg1*_ = *t*_1_ − *t*_2_ and the coupling coefficient *H*_*sg*2_ =  − (*t*_1_ *x*_1_ + *t*_2_ *x*_2_), as seen in Eq. (9). Figure 7 shows the impact of the two coupling coefficients on the density diffuseness and on the neutron-skin thickness. The present results show that density radii are not seriously affected by changing the two combinations *H*_*sg1,2*_. Most important, increasing the difference between *t*_1_ and *t*_2_ increases the proton (Fig. 7a) and neutron diffuseness, and slightly increases the neutron-skin thickness. The neutron diffuseness (Fig. 7b) remains increasing with increasing the coefficient of the total spin–orbit density part of the tensor term (*H*_*eff2*_), but the proton diffuseness and the neutron-skin thickness reverse their behaviour with *H*_*sg1*_. While *a*_*p*_ tends to decrease with increasing *H*_*eff2*_, *∆*_*np*_ (Fig. 7c) increases with it. The neutron diffuseness is then generally increases with increasing the tensor contribution of the EDF. The proton diffuseness (neutron-skin thickness) probably keep fixed (slightly increases) with increasing this tensor contribution, under the opposite effects of increasing its two parts on them.

### Saturation bulk NM properties related to the Skyrme EDF

**Figure 8:**
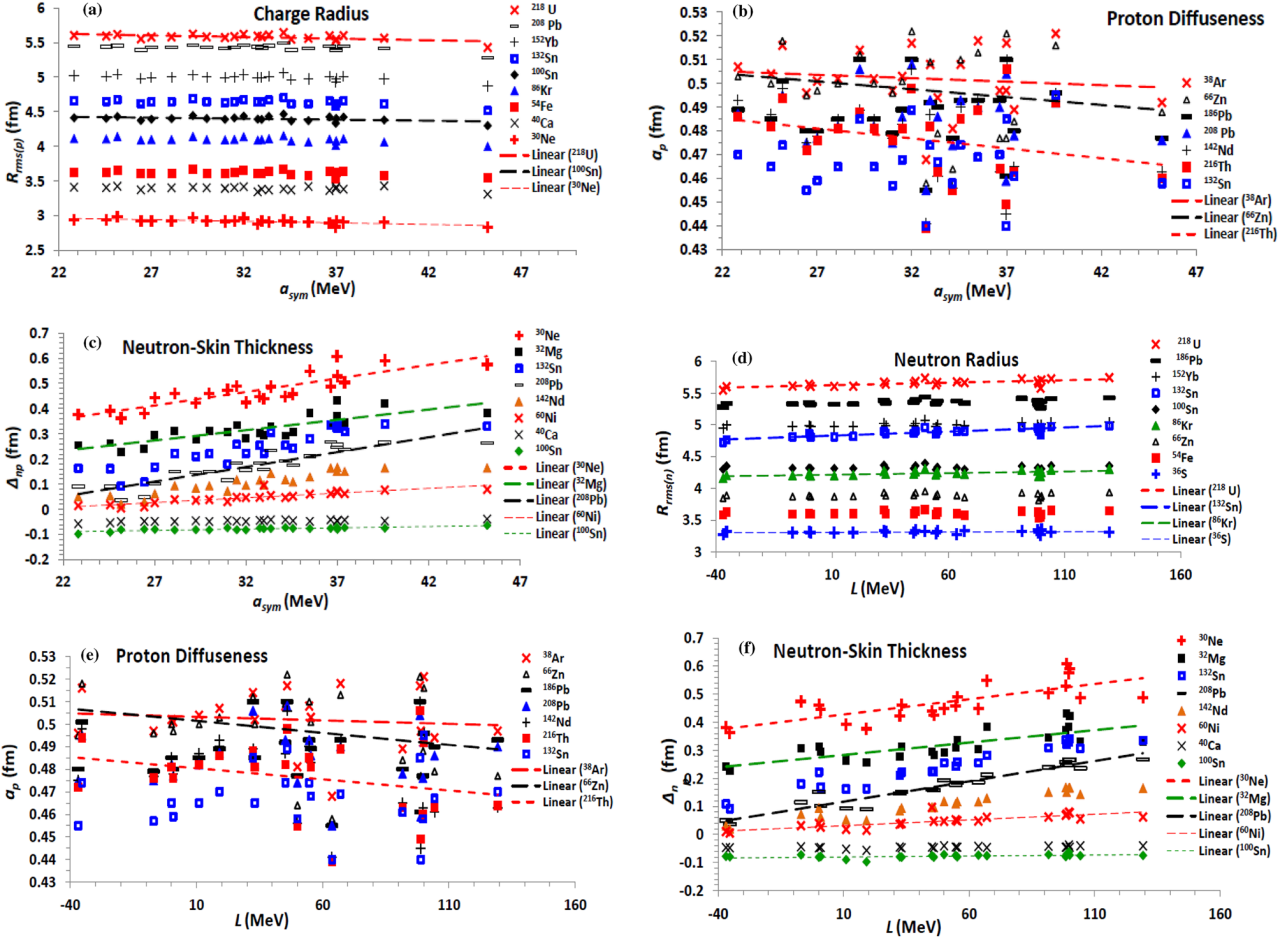
The (**a**) rms proton radius (**b**) proton diffuseness (**c**) neutron-skin thickness based on the considered Skyrme forces, as functions of the symmetry energy coefficient (*a*_sym_), and (**d**) the rms neutron radius (**e**) proton diffuseness (**f**) neutron-skin thickness as functions of the density slope (*L*) of the symmetry energy.

The considered EDFs of the effective NN interaction yield EOS characterized by NM symmetry energy 22.83 (SkSc10) ≤ *a*_*sym*_(MeV) ≤ 45.20 (SGOI), density-slope of symmetry energy − 36.86 (Es) ≤ *L* (MeV) ≤ 129.33 (SkI5), and incompressibility 201.69 (SkT5) ≤ *K*_*o*_ (MeV) ≤ 361.59 (SGOI) coefficients, and isoscalar effective nucleon mass 0.58 (SII) ≤ m*/m ≤ 1. Figure 8 shows the correlation between the proton and neutron density parameters and the coefficients of the symmetry energy (*a*_*sym*_) and its slope (L) at saturation density. The present results based on the different considered forces show that the charge radius of the investigated isotopes slightly increases with increasing both the symmetry energy (Fig. 8a) and its density- slope. On contrary, the radius of the neutron distribution tends to increase with increasing both *a*_*sym*_ and *L* (Fig. 8d). Figure 8b and e show that the diffuseness of the proton distribution decreases with increasing both quantities. The neutron diffuseness follows the same behaviour. The neutron-skin thickness is mainly governed by the symmetry energy. This clearly appears in Fig. 8c and f, which respectively show that *∆*_*np*_ increases with increasing the symmetry energy and its density-slope.

**Figure 9:**
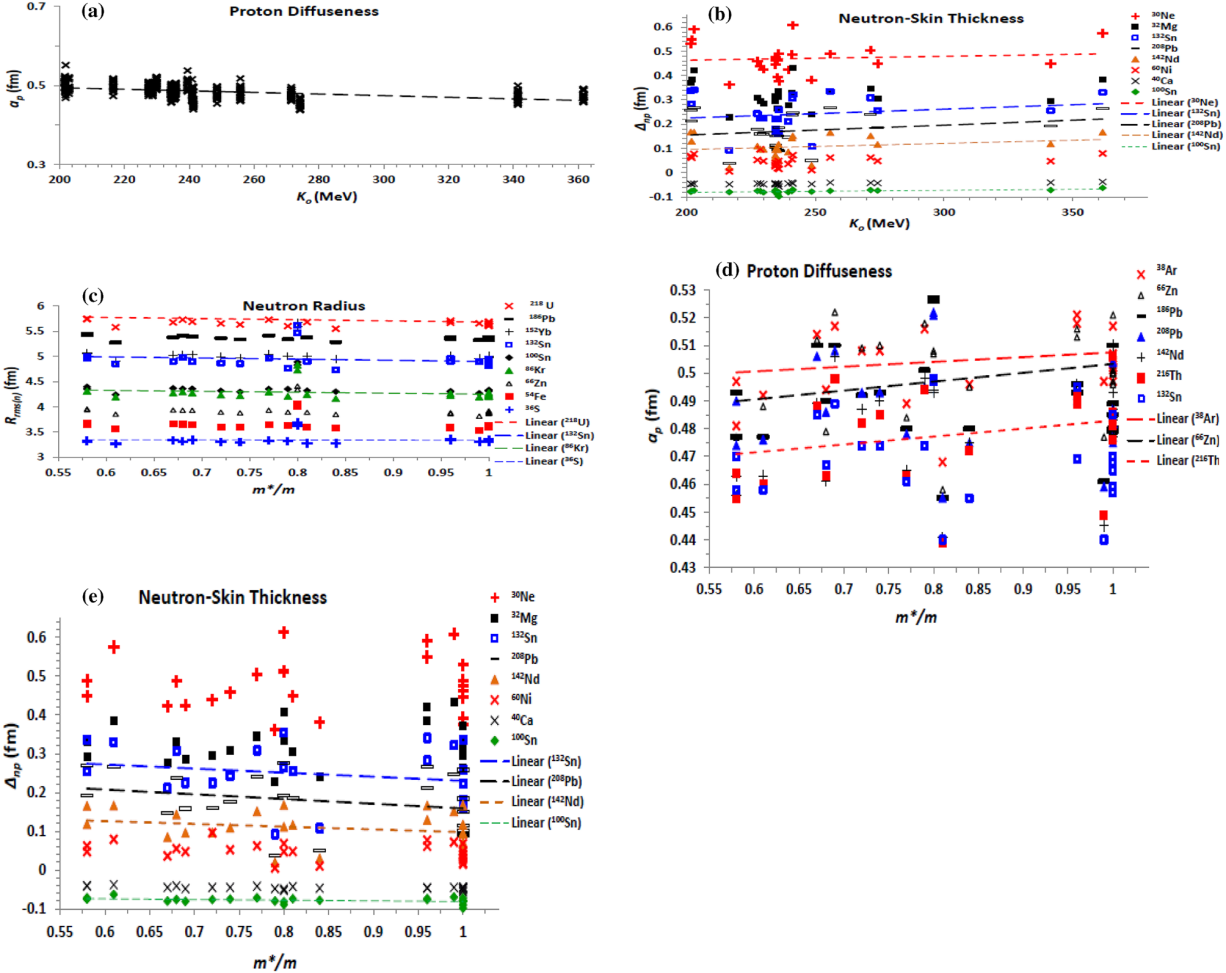
(**a**) The proton diffuseness for all investigated isotopes and (**b**) neutron-skin thickness for the isotopes mentioned on the panel, as functions of the NM incompressibility coefficient (*K*_o_) of the EOSs based on the considered Skyrme forces. (**c**) The rms neutron radius, (**d**) proton diffuseness, and (**e**) neutron-skin thickness of the mentioned isotopes, as functions of isoscalar effective nucleon-mass at saturation density, based on the considered Skyrme forces.

The incompressibility coefficient is the basic quantity which distinguishes the different EOSs of NM. The stiffer NM is characterized by a larger value of incompressibility coefficient. The dependence of the obtained diffuseness of the proton density and the neutron-skin thickness on the NM incompressibility coefficient of the EOS based on the used effective interaction is displayed in Fig. 9a and b, respectively. The obtained density profiles based on the considered effective forces generally show that the proton and neutron radii slightly decrease with increasing the stiffness of NM. As expected, the density diffuseness decrease with increasing the stiffness of NM, as seen from the behaviour of the proton diffuseness of the whole isotopes under investigation with *K*_*o*_ in Fig. 9a. Figure 9b shows that the neutron-skin thickness tends to increase with increasing the stiffness of NM. This also understood where the highly asymmetric nuclear matter at the surface region of the nucleus is softer than nuclear matter of less isospin asymmetry within the core of the nucleus. The stiffness of NM generally decreases with increasing its isospin asymmetry^1,4^.

The effect of the effective nucleon mass on the density distributions inside the nucleus is shown in Fig. 9c–e. As shown in Fig. 9c, the radius of the neutron distribution slightly decreases with increasing the effective mass. The proton radius follows the same trend. The proton and neutron diffuseness increases with increasing m*, as shown for the proton diffuseness in Fig. 9d. This expectedly reverses the behaviour of the density diffuseness with the effective mass term of the EDF shown in Fig. 4a and b, where the value of the isoscalar effective nucleon mass is given by the reciprocal of the combination 3*t*_1_ + *t*_2_(5 + 4*x*_2_) of the EDF parameters, as given by Eq. (18), which is similar to the combination *H*_*eff1*_ presented in Fig. 4a and b. As seen in Fig. 9e, increasing the effective mass decreases the neutron-skin thickness of the nucleus. Finally, we summarize in Table 1 the effects of the different terms of the EDF and of its related NM characteristic properties on the diffuseness of the proton and neutron density distributions of finite nuclei, and the corresponding neutron-skin thickness.

**Table 1:** The impact of increasing the different contributions of the Skyrme EDF of the NN effective interaction and their parameters, and of the bulk properties of the corresponding EOS of nuclear matter, on the proton and neutron diffuseness (*a*_*p,n*_), and on the neutron skin-thickness (*∆*_*np*_) of the density distributions of finite nuclei.

EDF term	Parameter/combination	*a*_*p*_	*a*_*n*_	*∆*_*np*_
Zero-range	***t***_***o***_	Decreasing (Dec)	Dec	Dec
	***x***_***o***_	Slightly decreasing (sl. Dec)	Almost fixed (al. Fix)	Increasing (Inc)
	Full term	Dec	Dec	Inc
Density-dependent	**σ**	Dec	Dec	Slightly increasing (sl. Inc)
	***t***_**3**_	Inc	Inc	sl. Inc
	***x***_**3**_	sl. Inc	sl. Dec	Dec
	***t***_**3**_*** x***_**3**_	sl. Inc	sl. Inc	Dec
Effective mass term	***t***_**1**_**(2 + *x***_**1**_**) + *t***_**2**_**(2 + *x***_**2**_**)**	Dec	Dec	sl. Dec
	***t***_**2**_**(2*x***_**2**_** + 1) − *t***_**1**_**(2*x***_**1**_** + 1)**	sl. Dec	Dec	sl. Dec
	Full term	Dec	Dec	Dec
Finite-range	**3*t***_**1**_**(2 + *x***_**1**_**) − *t***_**2**_**(2 + *x***_**2**_**)**	Inc	sl. Dec	sl. Dec
	**− [3*t***_**1**_**(2*x***_**1**_** + 1) + *t***_**2**_**(2*x***_**2**_** + 1)]**	Dec	Inc	Inc
	Full term	al. Fix	sl. Inc	sl. Inc
Spin–orbit	***W***_**o**_	Inc	Inc	Inc
J^2^ Tensor term	***t***_**1**_** – *t***_**2**_	Inc	Inc	sl. Dec
	**− (*t***_**1**_*** x***_**1**_** + *t***_**2**_*** x***_**2**_**)**	Dec	Inc	Inc
	Full term	al. Fix	Inc	sl. Inc
NM symmetry energy coefficient (*a*_*sym*_)		Dec	Dec	Inc
NM density-slope coefficient of symmetry energy (*L*)		sl. Dec	Dec	Inc
NM incompressibility coefficient (K0)		Dec	Dec	Inc
Isoscalar effective nucleon mass in NM (m*/m)		Inc	Inc	Dec

## Summary and conclusions

In this study, we have systematically investigated the influences of the different terms of the Skyrme NN effective interaction on the calculated proton and neutron distributions of finite nuclei, in context of the nuclear density functional theory. The influences of the different force terms and of their corresponding parameters are simultaneously analysed through different parameterizations of the EDF of the force. In particular, we performed the mean field calculations of the density distributions of eighteen even-even spherical nuclei, based on twenty five Skyrme force parameterizations. These different parameterizations are of broad ranges of their Skyrme parameters and of the corresponding NM symmetry and bulk properties, but they all successfully predict the ground-state properties of finite nuclei. The chosen nuclei also cover wide ranges of *Z*, *N*, *A*, and their isospin-asymmetry coefficient. We have found that the most sensitive density parameter to the various Skyrme force contributions and to their different parameters is the diffuseness of the density distributions, then the neutron-skin thickness. The diffuseness of both the proton and neutron density distributions is found to decrease upon increasing the central zero-range and effective mass terms, and the power σ of the density-dependent term of the effective force, but to increase with the spin–orbit strength, and with the combination (*t*_1_ − *t*_2_) of the tensor term. Increasing the combination coupled coefficient *t*_3_*x*_3_ of the density dependence term slightly increases the density diffuseness. Increasing the finite-range and the *J*^2^ tensor terms tend to slightly increase the neutron-skin thickness, remaining the proton diffuseness almost unchanged, due to the opposite effects of their parts. The neutron diffuseness increases (slightly increases) with increasing the J^2^ tensor (finite-range) term. The neutron-skin thickness also increases with the zero-range term and slightly increases with the power σ of the density-dependent term, but it decreases with increasing the effective mass term and the density-dependence combination coefficient *t*_3_*x*_3_. Moreover, the density radii show decreasing behaviour with increasing the zero-range and spin–orbit terms, but they show roughly fixed behaviour with increasing the density-dependent, the effective-mass, and the finite range terms. Generally, the terms and parameters that increase the attraction of the force decrease the radius of the density distribution, and indicate less diffuseness and larger internal density. The influences asymmetry and surface terms increases for the nuclei of larger isospin-asymmetry, displaying its minimum effect on the isospin-saturated isotopes. The individual effect of the EDF parameters on a certain nucleus ultimately depends on its isospin asymmetry, its shell closures, and on its size.

On the other hand, increasing the NM symmetry energy and its density-slope at saturation density decreases the neutron diffuseness, and slightly decreases (increases) the charge (neutron) radius. The proton diffuseness markedly decreases with increasing *a*_*sym*_, and slightly with increasing *L.* The corresponding neutron-skin thickness tends to increase upon increasing *a*_*sym*_, *L*, and *K*_*o*_, but it decreases with increasing the isoscalar effective mass. Increasing the indicated isoscalar effective mass also decreases slightly the density radii, but increases the diffuseness. Both the diffuseness and radii of the density distributions decrease with increasing the stiffness of the nuclear matter, which is indicated by the incompressibility coefficient. The correlations indicated in the present study between the calculated density distributions and the parameters of the different terms of the Skyrme effective NN interaction and the corresponding NM properties would help in resolving the ambiguity of any results based on these interactions, especially those related to the density distributions of the involved nuclei, such as the neutron-skin thickness and the isotopic shift, as well as the nuclear reaction and decay studies based on energy density formalism.

## Data Availability

All data generated or analysed during this study are included in this published article, or in the cited published articles.
